# Improving referral rates for smoking cessation: A multifaceted intervention aimed at radiation oncologists

**DOI:** 10.1016/j.tipsro.2023.100225

**Published:** 2023-11-25

**Authors:** Iris Bronsema, Luc van Lonkhuijzen, Peter Scholten, Janna Laan, Henrike Westerveld, Lukas Stalpers

**Affiliations:** a Department of Radiotherapy, Amsterdam UMC, University of Amsterdam, Location AMCMeibergdreef 9, 1105 AZ Amsterdam, the Netherlands; b Department of Gynaecology, Cancer Center Amsterdam, Amsterdam UMC, University of Amsterdam, Location AMC, Meibergdreef 9, 1105 AZ Amsterdam, the Netherlands; c Department of Radiotherapy, Erasmus MC Cancer Institute, University Medical Center Rotterdam, Rotterdam, the Netherlands

**Keywords:** Smoking cessation, Cancer, Radiotherapy, Referral, Consultation

## Abstract

**Objective:**

Continuation of smoking after a cancer diagnosis increases the burdensome side effects from cancer treatment, and decreases the chances of cure. Smoking cessation may improve oncological outcomes in cancer patients. This study aims to evaluate if radiation oncologists can be motivated by a smoking cessation awareness intervention to discuss smoking status more frequently and increase the referral rate for smoking cessation-support.

Study design and Setting:

A multifaceted approach was used to improve awareness: First, current practice was evaluated by a retrospective chart review of 282 patients referred for radiotherapy to ascertain smoking status, discussion of smoking cessation support and referral rates. Secondly, radiation oncologists were interviewed about their motives and barriers to discuss smoking status and smoking cessation support. The results were fed back in a teaching lecture to the doctors involved. Finally, the effect of this smoking cessation awareness intervention was prospectively evaluated in 100 patients.

**Results:**

After the smoking cessation awareness intervention, smoking cessation was more frequently discussed compared to baseline (77% (10/13)) and 39.5% (17/43) respectively. The referral rate for smoking cessation increased from 2.3% (1/43) to 69.2% (9/13)

**Conclusion:**

Without an active smoking prevention awareness policy, referral for smoking cessation support for cancer patients by radiation oncologists is low. A relatively short and simple smoking awareness intervention for radiation oncologist may result in a more frequent discussion with patients about smoking cessation and an even larger increase in referrals for smoking cessation support.

## Highlights

• Tobacco smoking is a major health problem and an important avoidable risk factor for many illnesses including cancer, cardiovascular-, and cerebrovascular illnesses.
• Without an active smoking prevention awareness policy, referral for smoking cessation support for cancer patients by radiation oncologists is low.
• A short and simple smoking awareness intervention for radiation oncologist may result in a more frequent discussion with patients about smoking cessation and an even larger increase in referrals for smoking cessation support.

## Introduction

Tweetable statement:

Referral rates for smoking cessation for patients starting treatment with radiotherapy can be improved with a simple intervention aimed at radiation oncologist.

Tobacco smoking is a major health problem and an important avoidable risk factor for many illnesses including cancer, cardiovascular-, and cerebrovascular illnesses. Worldwide tobacco use is responsible for 22 % of cancer deaths [1]. Smoking is the single most deleterious and preventable determinant of premature death in the Western World [2], [3], [4], [5].

A large number of patients with cancer undergoing cancer treatment are current- or previous-smokers. Smoking is not only a major cause of cancer, but may also give an up to two- to three-fold increase in cancer mortality compared to non-smoking patients, and an up to 10-fold increase of severe late complications of cancer treatment [2], [6], [7], [8]. Continued smoking increases the risk of side effects caused by radiotherapy. Smoking cessation reduces and sometimes normalizes these risks to the level of never smokers [8], [9], [10]. Despite the great progress made in supportive care for people with cancer, smoking cessation support remains an often-neglected element of cancer care [11], [12]. Many people continue to smoke even after a cancer diagnosis.

To help patients quit smoking before the start of cancer treatment physicians should address smoking behaviour, motivate patients to quit and arrange for referral to smoking cessation support. After a cancer diagnosis, the chance that a smoking patient will quit smoking is almost five times higher compared to the antecedent five years [8], [13]. This chance is even higher when patients follow a smoking cessation program [14]. Without cessation support, only 4 % of attempts to quit tobacco are successful. A combination of behavioural support and pharmacological intervention may double the chance of successful cessation [1]. Moreover, prolonged smoking cessation is more frequently seen after a recent oncological diagnosis [15]. Therefore, it is quite essential that oncological caregivers pay attention to smoking, and support their smoking patients with smoking cessation. In the Netherlands one of the quality criteria for cancer centres is that smoking status should be addressed with every patient [16].

Despite these good intentions, several recent studies suggest that smoking cessation interventions are as yet not well integrated into routine oncology practice, and that significant barriers prevent patients from accessing cessation services [17], [18]. Giuliani described five myths and the presumptions around smoking cessation in cancer: [18].

It is too late to quit smoking once someone already has cancer,

The time of diagnosis is not the right time to address smoking cessation,

Cancer patients are not interested in quitting smoking,

My patient is incurable; there is no role for smoking cessation,

It is not my job as an oncology practitioner/physician to address smoking cessation.

The authors stress the importance to recognize the harmful ways in which these myths influence health care providers’ willingness and likelihood to provide advice on smoking cessation and referral to services [17], [18].

To our knowledge only few studies are available about how and if smoking cessation is addressed in patients referred for radiotherapy. Day et al. [19] describes national data for radiation oncologist practice in Australia. They find that the large majority of oncologists record a smoking history, however only a small percentage enquire whether patients intend to cease tobacco use or advise cessation. A minority of oncologists then take action to facilitate patients quitting tobacco. We do not know how often smoking status and stop smoking interventions are discussed with cancer patients, and only few data are available of how many smoking patients are referred for a smoking cessation intervention

Although Dutch radiation oncologists are probably well aware of the risks of smoking, it cannot be excluded that Giuliani’s myths about smoking cessation prevail, thereby prohibiting more effective smoking cessation support in cancer patients. [12] In this study we investigated if a multifaceted approach aiming at improving awareness of smoking cessation among radiation oncologists resulted in more discussion of smoking status with their patients, and to more referrals for smoking cessation support.

## Methods

First, with the help of a retrospective chart review (baseline assessment), current practice was evaluated. Secondly, radiation oncologist were interviewed about their motives and barriers to discuss smoking status and smoking cessation support. The outcome of the baseline assessment and the interviews were fed back to the radiation oncologists and followed by a training in how to best address smoking status and on how to refer in a brief tutorial lecture. The effect of the intervention was assessed in a prospective assessment of 100 patients.

To evaluate the frequency in which smoking and smoking cessation was discussed, a retrospective (baseline assessment) chart review was performed in 282 consecutively included patients with all types of gynaecological cancer and different other common types of cancer who were referred for radiotherapy from January 1st, 2020 until June 1st, 2021 (Table 1). Cancer types with less than seven patients being treated in this study period were excluded. We analysed the notes of the first consultation by the radiation oncologist. The cohort was limited to patients who received radiotherapy as a part of curative treatment; patients treated with palliative intent were excluded.

For all included patients, data were extracted from the electronic patient chart and recorded into a case record form (CRF). The following items were recorded: if smoking status was discussed by the radiation oncologist, if it was recorded during first patient consultation, and if in case a smoking patient was referred for any type of smoking cessation intervention. Smokers were defined as patients who did still smoke on the day of their appointment. Recent quitters (<30 days) were included in the non-smokers group.

The myths and barriers that may inhibit smoking cessation referral were explored amongst radiation oncologists. After obtaining consent, 15 radiation oncologists were interviewed exploring awareness about the risks of smoking during and after radiotherapy. They were asked about their practice and potential barriers around discussing smoking status with patients. Furthermore, the radiation oncologists were interviewed about their knowledge of the possibilities for referring and efficacy of smoking cessation support. We explored presumptions around smoking cessation in cancer and we evaluated the practice implications for cancer care. We developed a structured interview with 14 questions, which we used in every interview (supplementary table 1).

The intervention was aimed at improving referral rates for smoking cessation. All radiation oncologists were offered a training session to improve awareness their current referral patterns and to inform them about the importance of addressing smoking status and possibilities for smoking cessation support. Because sessions were given during normal working days the content was condensed to be discussed within 20 min. During the session, the results of the retrospective baseline chart review were presented, the different myths and barriers of smoking cessation were discussed [18], and a practical instruction was given how and where to record smoking status in the electronic patient chart. A short explanation was given about the Very Brief Advice method. This method teaches health care workers to explore motivation and refer patients in a very limited time. It is a very simple intervention that is designed to be used opportunistically in less than 30 s in almost any encounter with a smoker [20]. There are three elements to Very Brief Advice: establishing and recording smoking status (ASK); advising on how to stop (ADVISE); and offering help or referral (ACT). Lastly it was explained to whom smoking patients can be referred to for specific stop smoking support. We offered the training to the complete department of radiotherapy of the AUMC between the 5th and the 9th of July 2021. The training session was repeated two times to maximize the opportunity for radiation oncologists to attend.

To evaluate the effect of the intervention, a prospective chart review was performed evaluating 100 patients who visited the outpatient clinic of the radiotherapy department at Amsterdam UMC from July 12th, 2021 until August 20th, 2021. We included patients with these same cancer types as the pre-intervention cohort. For the prospective cohort the same data as done in the retrospective study, were retrieved from the patient charts.

Pearson’s Chi-square test was used to compare baseline and post-intervention results. Patients were retrospectively and prospectively recruited under a waiver for informed consent from the Medical Ethical Board of the Amsterdam UMC – University of Amsterdam (ID Code: W19_199 # 19.245).

## Results

For the pre-intervention cohort, 282 patient were included who came for a first consultation at the radiotherapy department. Patient characteristics are summarized in Table 1**.** In 81.2 % (n = 229) of the patient charts, the smoking status was recorded, discrete or in plain text. Of these patients, 15.2 % (n = 43) were active smokers at the moment of their first appointment.

**Table 1:** Patient characteristics off all patients included in the retrospective cohort.

		**Smokers**		**Non-smokers**		**Unknown**		**Total**
		N=	%	N	%	N	%	N
**Gender**	Male	11	21.2	23	44.2	18	34.6	52
	Female	32	13.9	195	84.8	3	1.3	230
**Type of cancer**								
	Cervix	13	20.0	52	80.0	0	0	65
	Endometrium	6	7.1	79	92.9	0	0	85
	Vagina	2	15.4	11	84.6	0	0	13
	Vulva	2	16.7	10	83.3	0	0	12
	Bladder	2	28.6	3	42.8	2	28.6	7
	Lung	2	18.2	8	72.7	1	9.1	11
	Mamma	6	14.3	35	83.3	1	2.4	42
	Prostate	7	19.0	15	40.5	15	40.5	37
	Colon	3	30.0	5	50.0	2	20.0	10
**Age**								
	<30	1	100	0	0	0	0	1
	30–39	2	8.7	21	91.3	0	0	23
	40–49	2	11.1	16	88.9	0	0	18
	50–59	12	30.8	27	69.2	0	0	39
	60–69	15	16.5	66	72.5	10	11.0	91
	70–79	9	10.6	71	83.5	5	5.9	85
	80–89	2	8.0	17	68.0	6	24.0	25

During the first appointment, smoking cessation was discussed with only 39.9 % (17/43) of the smoking patients. One of the patients (1/43, 2.3 %) who smoked was referred for specific smoking cessation support by the radiation oncologist. In addition, 11.6 % (n = 5) of the patients were already referred for specific stop smoking support by another health care provider.

## Interviews

Fifteen radiation oncologists from the AUMC were interviewed about their awareness of the risks of smoking during and after treatment and about their knowledge of the possibilities and efficacy of stop smoking support.

Thirteen (87 %) of the radiation oncologists stated that they always discuss smoking status with patients during the first consultation and that they suggest smoking cessation. They discuss with their patients the amount of cigarettes they smoke and when they started smoking. Furthermore, the radiation oncologists informed their patients about the side effects of continuation of smoking during treatment with radiotherapy. Moreover, they indicated to speak with patients about the reduced oncological outcomes and increased toxicity of radiotherapy in case of smoking continuation.

Eleven (73 %) radiation oncologists indicated that they felt no barriers to discuss smoking and smoking cessation with patients. The remaining four (27 %) mentioned time as main barrier. Fourteen (93 %) knew to whom to refer patients for stop smoking support. During the interviews, a patient’s general practitioner and a specialised nurse of the radiotherapy department were most frequently mentioned by the radiation oncologists as options for a referral for stop smoking support. In the electronic chart smoking status can be recorded as a discrete data point. In this way the data about smoking can be easily found by other clinicians or retrieved and used for quality analysis or automatically generated treatment suggestions. Only twelve (80 %) of the radiation oncologists were acquainted with this way to report smoking status in the electronic chart, but, only five (33 %) used this discrete manner.

During the interviews, we asked the radiation oncologists for reasons to not report smoking status as a discrete data point. Several answers were given: ‘Because of a lack of time I do not report the smoking status in a discrete manner’; ‘It is not important for me to report smoking status in a discrete manner’; ‘I cannot find the information about smoking status in the electronic patient charter after I filled it in’; ‘I think it is not useful to report smoking status in a discrete manner’; ‘I have to note smoking status in different places in the charts which is needless in my opinion’; ‘most of the doctors do not care whether I report smoking status in a discrete manner or not’.

Eight (53 %) of the radiation oncologists indicated that they record their referral for specific stop smoking support in the letter to the general practitioner sent after the first consultation. Only three (20 %) indicated to be aware of the smoking cessation guideline in the hospital and two (13 %) indicated to be aware of the national guideline.

## Prospective study

From July 12th, 2021 until August 20th, 2021 100 patients were accrued: Patient characteristics are presented in Table 2**.**

**Table 2:** Patient characteristics of all patients included in the prospective chart review.

		**Smokers**		**Non-smokers**		**Not recorded**		**Total**
		N	%	N	%	N	%	N
**Gender**								
	Male	4	30,8	30	38	7	87,5	41
	Female	9	69,2	49	62	1	12,5	59
**Type of cancer**								
	Cervix	1	14.3	6	85.7	0	0	7
	Endometrium	0	0	13	100	0	0	13
	Uterus	0	0	0	0	0	0	0
	Vagina	1	33.3	2	66.7	0	0	3
	Vulva	0	0	0	0	0	0	0
	Mamma	5	19.2	21	80.8	0	0	26
	Prostate	3	10.0	20	66.7	7	23.3	30
	Colon/Anus	2	25.0	5	62.5	1	12.5	8
	Bladder	0	0		100	0	0	3
	Lung	1	10.0	9	90.0	0	0	10
**Age**								
	<30	0	0	0	0	0	0	0
	30–39	0	0	4	100	0	0	4
	40–49	1	12.5	7	87.5	0	0	8
	50–59	5	41.7	7	58.3	0	0	12
	60–69	4	10.0	33	82.5	3	7.5	40
	70–79	1	3.7	22	81.5	4	14.8	27
	80–89	2	22.2	6	66.7	1	11.1	9

Table 3 gives the results of the comparison of the retrospective (baseline) and the prospective (post-intervention) study. The recording of smoking status in patient correspondence or plain text did not change, but discrete recording did slightly decreased from 84.5 % to 74 % (p = 0.02). The proportion of active smokers did not change. But the proportion of patients in whom smoking cessation was discussed, increased significantly from 39.5 % to 76.9 % (p = 0.02). More so, the proportion of smoking patients who were referred for stop smoking support had increased from 2.3 % to 69.2 % (p < 0.001). The increase of referrals was higher in non-gynaecological patients than in gynaecological patients. However, in patients with prostate cancer, smoking status were only recorded in a minority of patients, but were virtually always recorded in breast cancer patients.

**Table 3:** Recording of smoking status and stop-smoking support in cancer patients by radiation oncologists before and after a smoking awareness training.

Description	Baseline assessment		Post-intervention assessment		OR (95 % Confidence Interval)	p-value
Number of patients	282		100			
Smoking status recorded in plain text	195	69.1 %	68	68.0 %	0.95 (0.58–1.55)	0.82
Smoking status recorded discrete	239	84.8 %	74	74.0 %	0.51 (0.29–0.89)	0.02
						
Active smoker	43	15.2 %	13	13.0 %	0.83 (0.43–1.62)	0.58
Smoking cessation discussed	17	39.5 %	10	76.9 %	5.10 (1.22–21.25)	0.02
Referred for stop smoking support						
- Relative to active smokers	1	2.3 %	9	90.0 %	94.0 (9.4–948.8)	<0.0001

OR = Odds Ratio, p-value = Fisher Exact Probability Test.

## Discussion

Radiation oncologists discussed smoking and smoking cessation more frequently with their patients after a smoking cessation awareness intervention. It resulted in a major increase in referrals for stop smoking support. Furthermore, the proportion of patient charts wherein smoking status were recorded decreased, although not significant.

Our study has some limitations. We did not include people treated with palliative intent to have a more homogeneous type of first encounter. In retrospect even we were inhibited by one of the barriers we mentioned above. Even for people treated for palliation smoking cessation has shown to be beneficial and they should not be excluded from smoking cessation advice.

A second limitation is the relative short post intervention follow up. We cannot excluded that the large difference in referral rates is temporarily in nature. However the intervention is limited in time and could easily be repeated in the future. Lastly we only included a limted number of smokers in the post intervention cohort.

When analysing how often smoking was recorded, we found differences between patients groups. This may be caused by a specific pre-formatted consultation template including a question about smoking which was available for patients with breast cancer showing a high registration rate, which was not included for patients with prostate cancer showing low registration rates. We recommend to include information about smoking status in all pre-formatted templates in the consultation notes, and include the explicit question if a smoking patient is referred for stop smoking support.

The higher rate of smoking behaviour recording as a discrete data point for patients with gynaecological cancer may be explained by the fact that stop smoking awareness had started earlier in the gynaecological oncology department. However, the proportion of smoking women with a gynaecological tumour, referred for stop smoking support was unexpectedly low. Although we did not explore the reasons for the low referral rate we expect that the same barriers play a role that are mentioned in the introduction such as lack of time, lack of awareness and low priority.

We found a remarkable discrepancy between the results of the retrospective chart review and the statements of the radiation oncologists during the interviews. The great majority of the radiation oncologists answered that they always discuss smoking status with patients during the first consultation and advise smoking cessation to smoking patients. However, the retrospective chart review showed that discussion of smoking cessation was recorded in only half of the patients with gynaecological cancer and only in one out of four patients with other cancers. However, during the interviews, some radiation oncologists mentioned that they discussed smoking cessation but did not always record this. Considering that the number of referrals for smoking cessation support increased, we hypothesise that increased awareness of smoking risk and options for referral made doctors realize better that they, as health professionals, have an essential contribution to guide patients toward healthier choices [21]. Previous research has demonstrated that health professionals may miss opportunities to advise cancer survivors about smoking cessation and/or assist them with cessation, or may not consider tobacco cessation treatment delivery as a core health care service [22], [23], [24], [25].

Reducing smoking among cancer survivors is a priority, given that cancer survivors are at increased risk for subsequent chronic diseases, recurrence of the cancer and that they have an increased risk on severe late effects when continuing smoking. Tobacco cessation among all cancer survivors can help improve prognosis, quality of life, and reduce the risk of further disease [13], [22].

One of the barriers the interviewed radiation oncologists mentioned was a lack of time in their outpatient clinic. To overcome this barrier the “Very Brief Advice” method was introduced during our training.

Aveyard concluded: ‘Both offering advice to stop smoking on medical grounds and support for cessation appear to increase the success rate of attempts to quit smoking’ [26], [27], [28]. It is more effective to promote smoking cessation support all smokers, also to smokers without willingness to quit, compared to only advise smoking cessation and refer to stop smoking support to those who are interested in smoking cessation [26]. Therefore, our advice to all health care providers is to use the ‘’Very Brief Advice’’ method in all smoking patients, regardless of their motivation to quit smoking.

During the interviews with the radiation oncologists, we gave recommendations to improve the awareness of smoking and smoking cessation. Many radiation oncologists suggested to make it easier to record smoking status in a discrete manner in the electronic health record. Furthermore, flyers with patient information about stop-smoking-support were requested by many radiation oncologists.

## Conclusion and recommendations

A brief and simple stop smoking awareness intervention for radiation oncologists greatly improves discussion of the risks of smoking and the benefit of smoking cessation with their cancer patients. Even in radiation oncologists who are knowledgeable about smoking, increasing awareness leads to a major increase in referrals for stop-smoking support. Lack of time in the consultation room was mentioned as the biggest barrier for radiation oncologists to discuss healthy lifestyles with patients.

## Funding

No funding was received for this study.

## Declaration of Competing Interest

The authors declare that they have no known competing financial interests or personal relationships that could have appeared to influence the work reported in this paper.
