# Supplementary material for: Improving referral rates for smoking cessation: A multifaceted intervention aimed at radiation oncologists
Source: Tech Innov Patient Support Radiat Oncol. 2023 Nov 25;29:100225. doi: 10.1016/j.tipsro.2023.100225 (PMC10761906; doi:10.1016/j.tipsro.2023.100225)
Supplement: Supplementary data 1 [file mmc1.docx]

Supplementary table; interview guide

1. Do you routinely ask patients about intoxications, especially smoking, during the initial interview? If yes, why do you? If no, why not?
2. If so, what exactly do you ask all in people who smoke
3. What are reasons/barriers for you to ask/not ask about smoking during the initial interview with a new patient?
4. What do you tell smoking patients about the consequences of smoking?
5. Do you discuss smoking cessation?
   1. How do you do that/what do you discuss?
6. Do you ask smoking patients if they are motivated to quit smoking?
   1. If you ask, do you also put this information in the letter/note?
7. What do you do, how do you approach it when people want to quit smoking?
   1. What steps do you take?
8. Do you know where to refer smoking patients when they want to quit smoking?
9. Do you know where in EPIC to record intoxications, especially smoking, appropriately (=discreetly)? (Can they show that?)
   1. Do you think this is important? Why yes/no?
10. Do you put the information about intoxications in the letter and in the note? How do you do this?
11. If you refer patients to a smoking cessation clinic, do you also put this in the letter?
12. Do you know the guideline/protocol that is in iprova?
13. Do you know the national guideline?
14. If you would like to improve smoking cessation care in our hospital/ within your department what do you think should be done?
